# Perspectives of people in Philadelphia who use fentanyl/heroin adulterated with the animal tranquilizer xylazine; Making a case for xylazine test strips

**DOI:** 10.1016/j.dadr.2022.100074

**Published:** 2022-06-30

**Authors:** Megan K. Reed, Nicholas S. Imperato, Jeanette M. Bowles, Venise J. Salcedo, Amanda Guth, Kristin L. Rising

**Affiliations:** a Department of Emergency Medicine, Sidney Kimmel Medical College, Thomas Jefferson University, 1015 Walnut Street, Curtis Building, Suite 704, Philadelphia, PS 19107, United States; b Center for Connected Care, Sidney Kimmel Medical College, Thomas Jefferson University, Philadelphia, PS, United States; c College of Population Health, Thomas Jefferson University, Philadelphia, PS, United States; d Centre on Drug Policy Evaluation, St. Michael's Hospital, Toronto, ON, Canada; e College of Nursing, Thomas Jefferson University, Philadelphia, PS, United States

**Keywords:** PWUD, people who use drugs

## Abstract

**Background:**

Xylazine is an animal tranquilizer increasingly detected in those who have died of an opioid overdose in Philadelphia, PA and elsewhere. Despite an increasing presence of xylazine in the local fentanyl/heroin drug market and its association with ulcers, there are few perspectives about xylazine from people who use drugs and no data about the utility of a hypothetical xylazine test strip.

**Methods:**

Between January to May 2021 in Philadelphia, PA, people who used fentanyl/heroin and had previously used fentanyl test strips were queried about xylazine and hypothetical xylazine test strips. Interviews were transcribed and analysis was conducted via conventional content analysis.

**Results:**

Participants (7 spontaneously, 6 after probing, *n* = 13) discussed “tranq” (i.e., xylazine) in the fentanyl/heroin supply. None enjoyed tranq or wanted it in their fentanyl/heroin. Participants suspected xylazine saturation of the fentanyl/heroin market, disliked the sensation of the drug, and had safety concerns about xylazine exposure. Participants did not indicate concerns about overdose. All were interested in hypothetical xylazine test strips. While previous literature indicates that some people enjoy tranq in their fentanyl/heroin, our findings differed, with participants expressing concern about the consequences of undesired exposure. The interest expressed for xylazine test strips by people who use fentanyl/heroin is an important opportunity to center their voices in the development of innovations designed to mitigate the harms of unwanted adulterant exposure.

**Conclusions:**

In the present study, people who use fentanyl/heroin indicated an interest to test their drug for the presence of xylazine prior to use.

## Highlights

• Xylazine, a veterinary tranquilizer, is increasingly in heroin/fentanyl in Philadelphia.
• While some people like the combination of these drugs, others may not.
• Our sample disliked the feeling of xylazine and a perceived risk of violence.
• Our sample expressed a desire to use xylazine test strips if they existed.
• These test strips would meet the needs of some people who use heroin/fentanyl.

## 1 Introduction

People who use drugs (PWUD) from the unregulated drug market have little control over the presence of unwanted substances within the drug products they obtain. Adulterants (e.g., fentanyl, caffeine) and diluents (e.g., mannitol, glucose) are often added to the unregulated drug supply, introducing risk for adverse health outcomes such as soft tissue infections, overdose, and other effects of drug poisoning (e.g., psychosis from synthetic cathinones, renal dysfunction from levamisole) (Griffin et al., 2022; Kariisa et al., 2021; Nolan and Jen, 2015; Oliver et al., 2019; Thangada et al., 2021). Xylazine, an animal tranquilizer, has been increasingly detected in the unregulated drug supply throughout the eastern United States (U.S.), Puerto Rico, and Canada (Bowles et al., 2021; Ruiz-Colón et al., 2014; Tobias et al., 2020; Torruella, 2011; Wong et al., 2008). Its presence in national overdose deaths was five times higher in 2020 than in 2015 and has been detected in 91% of fentanyl/heroin samples in Philadelphia (Friedman et al., 2022; Philadelphia Department of Public Health, 2022). Additionally, hospital-based toxicology screens of people in Philadelphia found that 78% of people screening positive for fentanyl also screened positive for xylazine (Korn et al., 2021). Xylazine is an alpha-2-adrenergic agonist that functions similarly to clonidine, which is a medication indicated to treat hypertension or occasionally used “off-label” to treat opioid withdrawal. It acts to decrease the release of excitatory neurotransmitters such as norepinephrine and dopamine in the central nervous system (CNS) and peripherally acts as a vasoconstrictor. This can lead to sedation, muscle relaxation, respiratory depression, hypotension, bradycardia, and decreased response to painful stimuli (Shi et al., 2016). The U.S. Federal Drug Administration has only authorized xylazine for veterinary use as a tranquilizer, leading to its colloquial name of “tranq” or “tranq dope” (Mohr et al., 2020).

Xylazine was first identified as an adulterant in heroin products two decades ago on the U.S. island territory of Puerto Rico (Torruella, 2011), where it is known as "anestesia de caballo" (horse anesthesia), and remains especially prevalent in speedballs (i.e., a mixture of heroin and cocaine). In one study, over 90% of speedball preparations tested from Puerto Rico were found to contain xylazine (Rodríguez et al., 2008). Early literature from Puerto Rico indicated that xylazine is linked to infected ulcerating skin lesions that are exacerbated by heroin/xylazine injection directly into the ulcer in attempt to reduce pain (Torruella, 2011). Recent reports have indicated that xylazine is increasingly being found in supplies of fentanyl/heroin and has similarly been linked to painful ulcers when injected (Johnson et al., 2021). The earliest reported identification of xylazine being utilized as an adulterant in Philadelphia is from April of 2006, in which a series of seven people who died from apparent overdoses were found to have urine samples positive for heroin metabolites, fentanyl and xylazine (Wong et al., 2008). Between 2010 and 2015 in Philadelphia, xylazine was detected in 2% of unintentional fatal overdose cases, which increased to 31% just four years later in 2019. Last year, the Frederic Rieders Family Foundation released a public health alert regarding the recent uptick in the presence of xylazine in heroin supplies (Mohr et al., 2020). They reported that xylazine produces a more potent high than heroin alone, potentiates and prolongs the effects of cocaine or opioids, and may increase the risk of opioid-related overdose or death. Additionally, the sedating effects of the drug places PWUD at increased risk of sexual assault (Andresen-Streichert et al., 2017).

Drug-checking using liquid reagents and other technology has been accessed by people who use drugs since at least the 1990s (Maghsoudi et al., 2022). A more rudimentary drug checking approach is fentanyl test strips, which are a low-barrier harm reduction method to test a drug for the presence of fentanyl before use. Research indicates that the use of fentanyl test strips is associated with greater adoption of safer drug use practices (e.g., using less of a drug at once, using with other people) (Peiper et al., 2019). Considering the utility of fentanyl test strips, a similar innovation could be useful for other adulterants in the unregulated drug supply, like xylazine. The present paper reports on such an iteration of fentanyl test strips identified by people who use fentanyl/heroin.

## 2 Methods

This is an analysis of qualitative data from a study to assess behavior outcomes among people who had previously used fentanyl test strips. We conducted qualitative interviews between January to May 2021 with 29 people in Philadelphia, PA who had previously used fentanyl test strips on a drug other than heroin. Two recruitment approaches were undertaken to generate the sample. First, team members used convenience sampling to recruit participants outside of a local harm reduction agency. Then, snowball sampling was employed based on referrals from participants.

Eligibility criteria included that individuals be 18 years old or older, able to understand English, and reported ever using a fentanyl test strip on a drug other than heroin. Participants who were eligible, provided verbal consent, and completed an interview were compensated with $20 cash. A semi-structured interview guide was developed that covered domains including fentanyl test strip practices, facilitators and barriers to fentanyl test strip use, and harm reduction actions based on fentanyl test strip use. Interviews were conducted by a qualitative researcher (MKR) and two research assistants experienced in conducting qualitative interviews (VJS, AG). Interviews were conducted in semi-private public locations selected by participants (e.g., a quiet street, an alley) or over the phone depending on participant preference and availability. Interviews were audio-recorded and transcribed verbatim.

PWUD were interviewed regarding their previous use of fentanyl test strips on drugs other than heroin (e.g., cocaine, synthetic cannabinoids, methamphetamine, street-acquired benzodiazepines). Twenty-one participants indicated active fentanyl/heroin use and one had used within the past year but reported “recently” ceasing fentanyl/heroin use. Over the course of these interviews, 13 of those 22 participants discussed the presence of “tranq”, or xylazine, in the local fentanyl/heroin supply. The topic of xylazine test strips arose organically among seven participants and probes about participant perspectives on hypothetical xylazine test strips were added to the interview guide for the last six qualitative interviews. Three coders (MKR, VJS, AG) analyzed all 13 of these transcripts using NVivo qualitative software. Analysis was guided by a conventional content analysis approach, in which codes are drawn directly from interview text, to identify themes (Hsieh and Shannon, 2005). This research was approved by the Institutional Review Boards at Thomas Jefferson University and the Philadelphia Department of Public Health. Quotes are presented using participant-selected pseudonyms, their age, gender, and race.

## 3 Results

Among 13 participants,76.9% were male (*n* = 10) and 84.6% were White (*n* = 11). All participants reported at least one previous overdose (see Table 1). Seven participants raised the topic spontaneously, while the remaining six were asked various questions to assess their viewpoints. None of the 13 participants wanted xylazine in the fentanyl/heroin that they were using.

**Table 1:** Demographics of Participants Discussing Experiences with Xylazine in Philadelphia, PA (*N* = 13).

**Characteristic**	**n (%)**
Age – mean (range), SD	41 (29–56), 7.9
Gender Identity	
Male	10 (76.9)
Female	3 (23.1)
Ethnicity	
Not Latino	11 (84.6)
Latino	2 (15.4)
Race	
White	11 (84.6)
African-American/Black	2 (15.4)
Previous Overdose from Any Drug^a^	13 (100.0)
Fentanyl/heroin Overdose	11 (84.6)
Cocaine Overdose	2 (15.4)
Synthetic cannabinoids (K2) Overdose	1 (15.4)
How Learned About FTS	
Harm Reduction Organizations	25 (86.2)
Peer/Friend	3 (10.3)
Police During an Arrest	1 (3.4)

a Some participants overdosed on different substances on separate occasions; numbers will not sum to 100%.

Common themes regarding suspected xylazine exposure included participants having an overwhelmingly unpleasant sensation when used or not lasting long enough to be worth the expense.

Because see, you got a lot of people that like tranq and fentanyl mixed together. And I told them, I said, I don't see how anybody can enjoy their high like that. I said, because all it does is knock you out. I said, to me, that's a waste of money. Yeah, because they're – that's – they figure it's a better high. It's a more intense high. But it's a waste of money because it don't hold you for jack. Don't have legs whatsoever. Don't have no kinda legs. (“E”, 56, male, Black)

You know, I don't like falling asleep and waking up and having no idea how I ended up on the floor or why my arm is sore from, you know, being in an awkward position or why I have cuts and bruises and I don't know how I got them. (“Shook”, 38, male, Latino)

Others elaborated on a fear of losing consciousness and awareness for extended periods, as noted by “Michael” (43, male, White):

I'm having more problems with the tranq then I am with the fentanyl down here. I have basically blackouts from the tranq. I lose days at a time. Like I'll lose four, five, six hours, and then when I come out of the blackout, my girlfriend's furious at me. She's crying. She wanting to leave me. And I have no idea what I've done for the last four or five hours.

Such social repercussions for unconsciousness or drug-induced parasomnia were common and, in some cases, violent. Participants discussed attitudes towards tranq within their communities, reporting that dislike of the drug was common. One participant said he would resell fentanyl/heroin that looked “tranqy”. E, who said the tranq did not “have legs”, elaborated on some of the risks of xylazine intoxication but acknowledged that some like the sensation.

And they hate when they get it by accident because, a lot of times, bad things will happen to them. They'll pass out somewhere, get robbed, won't remember what happened. All kinda crazy stuff. Because there's a lot of people out here that love tranq. I don't know why because it literally puts you out and then that's when people rob your stuff. (“E”, 56, male, Black)

Other participants noted the intensely unpleasant experience of withdrawing from what they believed were tranquilizers and the outdated treatment protocols at detoxification programs to appropriately treat both opioid and tranquilizer withdrawal.

Because it's like one of the worst detox right now, because the rehabs can't seem to find something to help with the – I guess they're putting tranquilizer in all of these bags too that they're selling out here. And the tranquilizer is the worst habit to kick because apparently it takes two to four weeks to get off of it. (“Nicole”, 29, female, White)

While not initially an identified topic of interest, multiple participants made statements regarding theoretical xylazine test strips. Nine participants discussed a desire to use such test strips if they existed, reporting that they would use them if available. For example:

I don't know if anyone has mentioned this, but the dope that they've been putting out here has tranquilizer in it too, and that's what's putting a lot of – that's what's killing a lot of people too. It is not necessarily the fentanyl but the tranq too. I don't know if there is a way for them make tranquilizer test strips too, but they would be useful to us in particular if they could make them too. (“Judah”, 32, male, White)

Some participants conveyed to the interviewer their belief that xylazine test strips would be more beneficial than fentanyl test strips (“I wish they would make a strip for [xylazine], to be honest with you." [“Nicole”]), Another said she would use xylazine test strips in combination with fentanyl test strips if both were available:

I definitely would [test for xylazine] because a lot of times I think like if you just fall asleep, I'm thinking it's like Seroquel or antidepressants. So I would definitely test both of them. See if either [fentanyl or xylazine] are in there. Or am I doing fake drugs all together? (“Kelly”, 39, female, White)

A few participants believed they could utilize fentanyl test strips to determine if their fentanyl/heroin contained xylazine. The belief was that if the fentanyl test strip was negative, then the heroin likely contained xylazine, and if the fentanyl test strip was positive, then xylazine was not present. Two participants specifically reported doing this if they sensed that the drug they purchased looked like it had either fentanyl or xylazine. For example, “Nick” (44, male, White), who believed he would use xylazine test strips more than fentanyl test strips, said:

I ask friends of mine that I see down there, if – what's good? Somebody telling me, I just try to test it to see, make sure it's not all tranq and that it comes up positive for fentanyl … Honestly, I believe it all has tranq in it, it's just the amount.

One participant noted that this approach was likely was not a reliable method to rule out adulteration with xylazine if the fentanyl test strip was positive.

Yeah.  But I think – I don't think that's an accurate – I think you could have both (fentanyl and xylazine). And just because it has fentanyl in it doesn't mean it's not gonna have tranq.  Because there's people out here that literally say that they have – that their dope has both. (“Tyler”, 37, male, Latino)

## 4 Discussion

Participants uniformly did not desire the presence of xylazine in their fentanyl/heroin but had limited to no control over xylazine or other adulterants in an unregulated drug market. They discussed concern about the increased vulnerability to which a mixture of fentanyl, heroin and xylazine potentially exposed them. They also noted disliking the sensation of the mixture of substances and worried about the implications of tranquillizer dependence. This concern was noted in recent ethnographic research, though authors of that study also recorded discussions about the extreme skin lesions associated with xylazine injection (Friedman et al., 2022). Our findings run counter to other work finding that some PWUD enjoy xylazine, especially as it can lengthen the duration of fentanyl and the combination can facilitate an enjoyable euphoria (Friedman et al., 2022). However, there was acknowledgement by participants that some people who use fentanyl/heroin in Philadelphia enjoy xylazine. But our sample expressed a strong dislike for the embodied experience of the combination and the sedating effects. Given that all were utilizing fentanyl test strips, people in this sample may be seeking a different use experience than those not using fentanyl test strips. More research is needed about preferences and practices related to xylazine consumption.

Available literature points to xylazine being viewed as a “drug of choice” in certain settings, including U.S colony Puerto Rico (Reyes et al., 2012). People who use drugs from Puerto Rico are often “relocated” by local governments from their homes in Puerto Rico to Philadelphia and other areas of the United States to receive largely unregulated drug treatment services (Torruella, 2012). Importantly, the adulteration of xylazine in heroin products in Puerto Rico is similarly out of the control of people who use drugs there. An adjustment to the enjoyment of its effects may have occurred over time. In Philadelphia, alongside a shifting unregulated drug market, there is potential that this “treatment relocation process” from Puerto Rico to Philadelphia has contributed to the local rise of xylazine as an adulterant in fentanyl/heroin. This could be explained, in part, by a growing community of “relocated” people who use drugs from Puerto Rico who have been accustomed to xylazine in heroin products for over two decades. While xylazine appears to have particularly been part of the Philadelphia fentanyl/heroin supply and likely the “epicenter” of the fentanyl/heroin/xylazine combination, it is increasingly being detected in other areas of North America (Bowles et al., 2021; Friedman et al., 2022; Tobias et al., 2020).

Xylazine is currently not a scheduled substance under the United States Controlled Substances Act, though some efforts are underway to change this (Drug Enforcement Administration, 2021; Murphy, n.d.). However, supply side efforts to control xylazine adulteration of fentanyl/heroin are unlikely to work and – similar to trends seen when trying to decrease the availability of alcohol, cannabis, and cocaine – will likely exacerbate adulteration (Cowan, 1986). Xylazine test strips, by contrast, are a demand-driven response to unwanted adulterants and may be able influence the composition of the drug supply if xylazine is linked to specific stamps (i.e., how fentanyl/heroin products are branded in Philadelphia) (Friedman et al., 2022). This new form of drug checking represents a potential tool to further empower PWUD to make informed choices about what and how they consume drugs.

All participants who spontaneously discussed wanting xylazine test strips, or were asked if they would want them, indicated they would use them to test their fentanyl/heroin before drug consumption, if available. Xylazine test strips are not currently available and, to our knowledge, are not in development. Research is needed from broader monitoring and analysis of the drug supply to determine whether xylazine in fentanyl/heroin is pharmaceutical grade. Additionally, it is important to understand if a xylazine test strip would be capable of detecting any xylazine analogs.

A xylazine test strip may have the potential to positively impact drug use in a similar manner to fentanyl test strips. Fentanyl test strips have been found to significantly alter drug use behavior and foster safer drug use practices with continued testing. Individuals using fentanyl test strips prior to drug use did so in order to prevent fentanyl overdose and the potential need for emergency interventions (Peiper et al., 2019). Additionally, there have been studies reporting fentanyl test strip use following drug use. Among these individuals, positive results for fentanyl were associated with use of reduced doses on subsequent drug consumption occasions (Karamouzian et al., 2018).

Our sample was comprised of people already using fentanyl test strips, indicating that distributing xylazine test strips along with fentanyl test strips may be an effective approach. People who use fentanyl/heroin may exhibit similar patterns with xylazine test strips: to determine if a particular use experience was related to xylazine exposure, to confirm the presence of desired xylazine, or to detect unwanted xylazine and alter subsequent actions as a result. Our previous research found that few people experience barriers to fentanyl test strip use and wish to receive them from a variety of locations; similar results may be seen among people if they had access to xylazine test strips (Reed et al., 2022).

Behavior change based on a positive xylazine test strip result has implications beyond overdose prevention efforts. The sedating effects of xylazine present a risk for victimization once used (Krongvorakul et al., 2018). Xylazine is also associated with an increase in severely infected ulcerations, often leading to excessive skin breakdown and necrosis, and in some cases resulting in amputations (McNinch et al., 2021). Xylazine test strips have the potential to mitigate these risks especially if xylazine-specific harm reduction approaches are known and resources to achieve them are accessible.

In addition to the development of xylazine test strips, there is an urgent need for harm reduction messaging to people who use fentanyl/heroin to mitigate the risks of unwanted exposure to xylazine. These should include safety planning in the event of heavy sedation, wound prevention, and wound care. Training should be provided to people who use drugs who may be exposed to xylazine and peers who can potentially provide guidance on naloxone administration in the event of an opioid overdose alongside xylazine ingestion. Moreover, guidance for addressing safety risks while the person is sedated is critical. Such guidelines should be developed in partnership with people who use drugs and field-tested for feasibility and acceptability, both in xylazine-saturated markets (e.g., Philadelphia) and elsewhere. The presence of safe consumption sites would present a safe place for someone heavily sedated after xylazine use. Finally, formal programs conducting point of care drug checking with spectroscopy technology could screen samples for the presence of expected and unexpected drugs, including xylazine

This study has limitations. The interview guide was not designed to ask participants about xylazine. As such, participants were not systematically asked about xylazine and the resulting sample size is small. Importantly, this sample was already using fentanyl test strips, indicating a bias towards using drug-checking technologies and may not represent the views of people not currently using fentanyl test strips. However, we believe these unanticipated results have important implications for the development of xylazine test strips to be responsive to drug market developments and the expressed wishes of people who use fentanyl/heroin. We did not probe about the specific route of administration for fentanyl/heroin use, though most referenced injection. Participants who used in other ways (e.g., insufflating) may have different risk profiles from participants who inject. Participants were predominantly White and male. Other demographic groups may have different perspectives. For example, women may have deeper concerns about the risk of sexual assault while under the heavily sedating effect of a heroin, fentanyl, and xylazine combination. Finally, this study took place in Philadelphia, Pennsylvania which appears to have a particularly high rate of xylazine adulteration of the fentanyl/heroin supply. While reports of increasing xylazine adulteration are emerging elsewhere (Bowles et al., 2021; Evans et al., 2021; Friedman et al., 2022; Nunez et al., 2021), the resulting concerns of people who use fentanyl/heroin in other locations may not be as prevalent.

## 5 Conclusion

The use of test strips as a harm reduction strategy is promising, but test strips for xylazine are not commercially available. If developed, they would likely be used by people who use fentanyl/heroin. It is important to be responsive to the stated needs of PWUD and consider allocation of resources to the development of xylazine test strips.

## Contributions

MKR was a major contributor to the design of the study, collected and analyzed data, and was a major contributor in writing the manuscript. NI was a major contributor in writing the manuscript. JMB interpreted the data and was a major contributor in writing the manuscript. VJS and AG collected and analyzed data. KLR made substantial contributions to the design of the study and was a major contributor in editing of the manuscript.

## Declaration of Competing Interest

Nothing to declare.
